# Identifying and characterising promising small molecule inhibitors of kinesin spindle protein using ligand-based virtual screening, molecular docking, molecular dynamics and MM‑GBSA calculations

**DOI:** 10.1007/s10822-024-00553-5

**Published:** 2024-04-01

**Authors:** Samia A. Elseginy

**Affiliations:** Chemical Industries Research Division, Green Chemistry Department, National Research Centre, Cairo, 12622 Egypt

**Keywords:** Similarity search, Ligand-based virtual screening, Kinesin spindle protein Eg5, Molecular dynamics, Binding free energy

## Abstract

The kinesin spindle protein (Eg5) is a mitotic protein that plays an essential role in the formation of the bipolar spindles during the mitotic phase. Eg5 protein controls the segregation of the chromosomes in mitosis which renders it a vital target for cancer treatment. In this study our approach to identifying novel scaffold for Eg5 inhibitors is based on targeting the novel allosteric pocket (α4/α6/L11). Extensive computational techniques were applied using ligand-based virtual screening and molecular docking by two approaches, MOE and AutoDock, to screen a library of commercial compounds. We identified compound 8-(3-(1H-imidazol-1-ylpropylamino)-3-methyl-7-((naphthalen-3-yl)methyl)-1H-purine-2, 6 (3H,7H)-dione (compound 5) as a novel scaffold for Eg5 inhibitors. This compound inhibited cancer cell Eg5 ATPase at 2.37 ± 0.15 µM. The molecular dynamics simulations revealed that the identified compound formed stable interactions in the allosteric pocket (α4/α6/L11) of the receptor, indicating its potential as a novel Eg5 inhibitor.

**Graphical Abstract:**

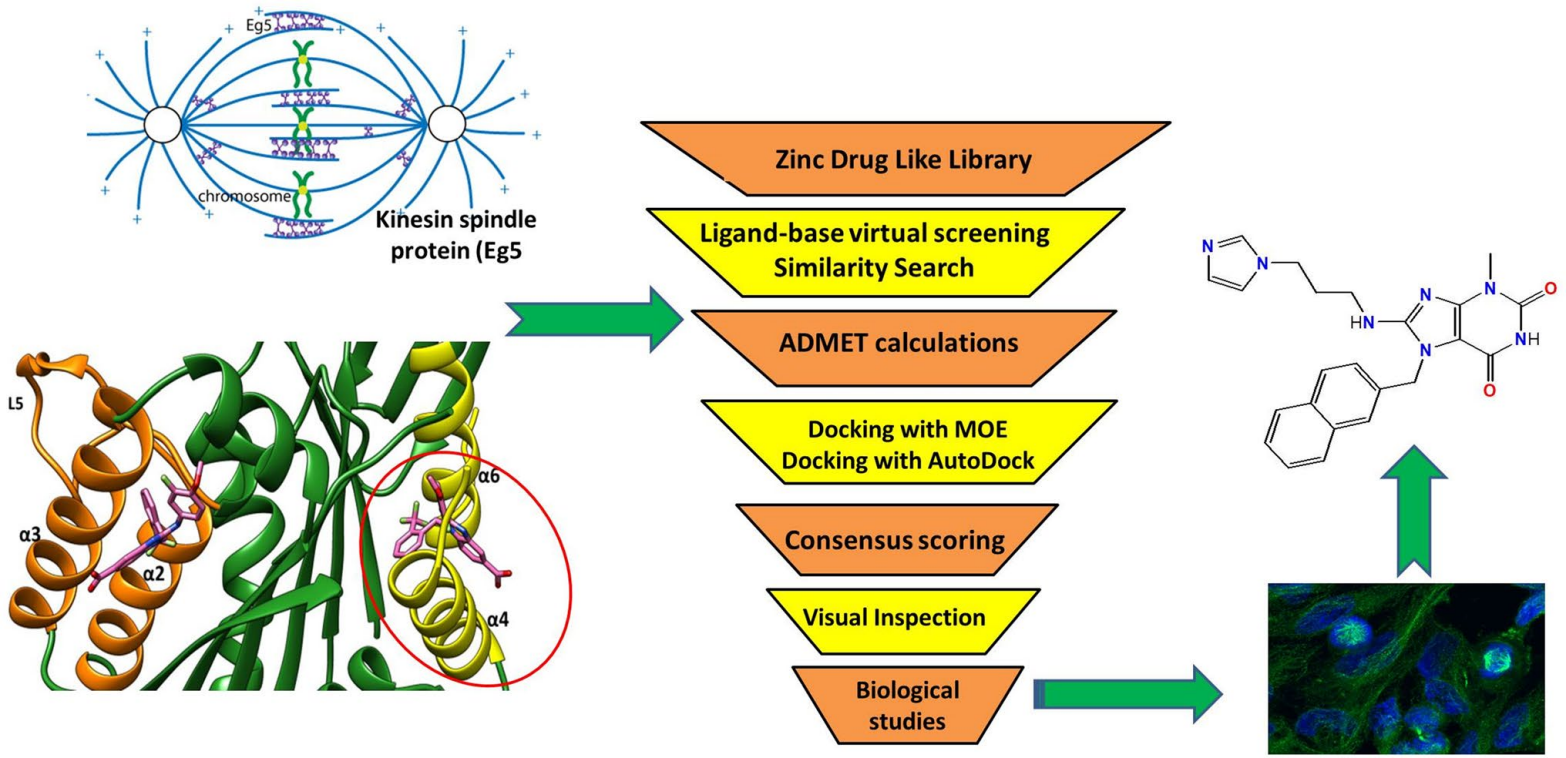

**Supplementary Information:**

The online version contains supplementary material available at 10.1007/s10822-024-00553-5.

## Introduction

Kinesins are a family of motor proteins that are responsible for a range of physiological functions, such as chromosome segregation, mitotic spindle assembly and vesicular trafficking [1]. Kinesins also called **‘‘**nanomotors”, because they generate energy from ATP hydrolysis and use it in the transport and movement of the intracellular cargos along the spindle microtubules (MTs) [2]. Eg5, also known as KIF11, is a member of the kinesins superfamily, particularly the mitotic kinesin-5 subgroup [3]. Eg5 is a homotetrameric motor protein that plays an important role in early mitosis through separation of the duplicated centrosomes, bipolar spindle formation, alignment and segregation of the chromosomes [4]. Eg5 was found to be overexpressed in various proliferative tissues, and was detected in many cancers, such as lung, pancreatic, breast, ovarian, bladder cancers and leukaemia, which renders Eg5 a potential target for developing novel inhibitors as anti-cancer agents [5, 6]. Eg5 inhibitors prevent centrosomes separation and mitotic spindle formation, which results in the formation of “monoasters” or monopolar spindles, and this causes mitotic arrest and the inhibition of cell division [1].

**Fig. 1:**
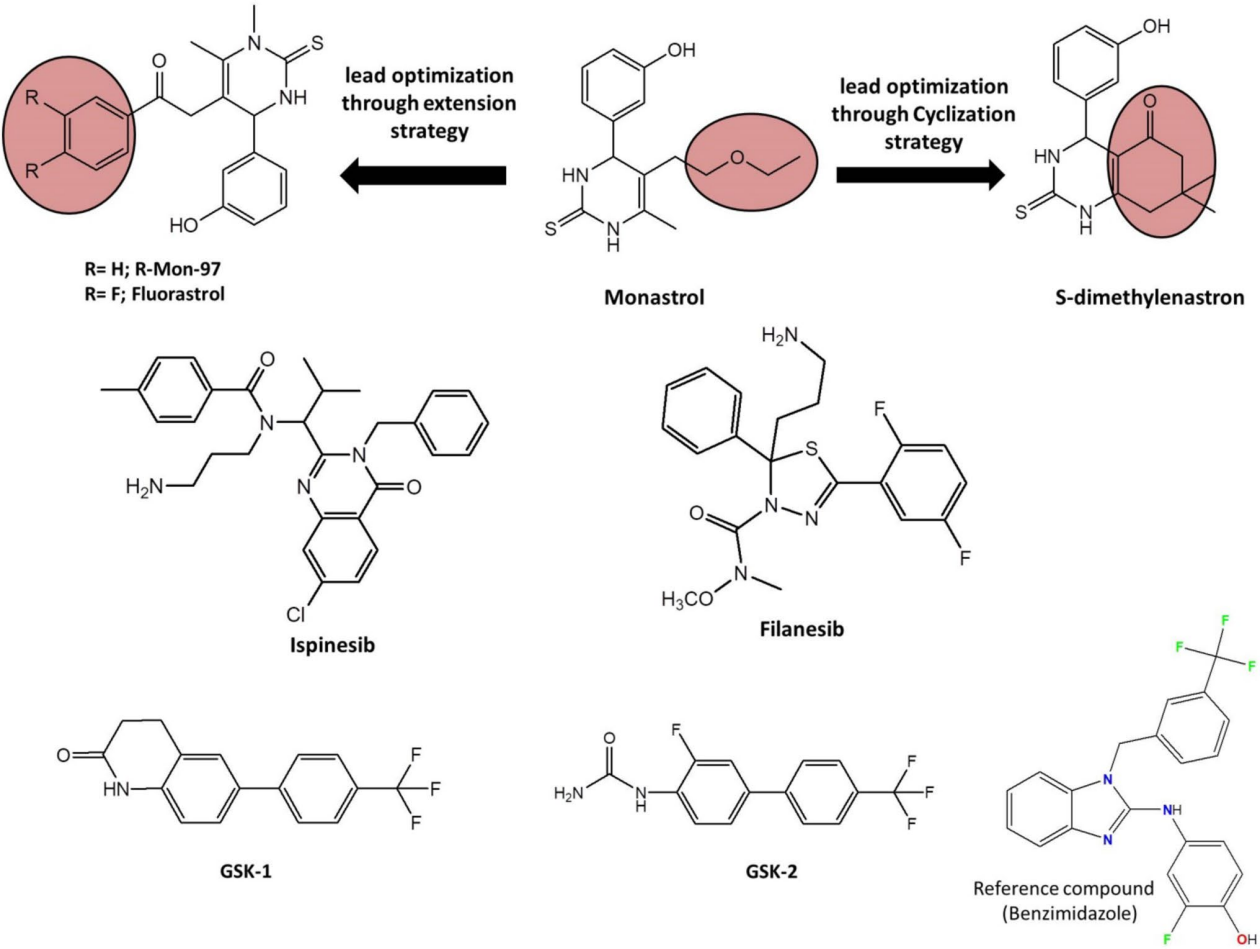
Chemical structure of Eg5 inhibitors

It was found that Eg5 inhibition in the human xenograft models, causes cell death and demonstrates anti-cancer activity [7]. Eg5 inhibitors are characterised by their selectivity and safety, because Eg5 functions only during mitosis; and does not affect the non-proliferative cells, which renders Eg5 inhibitors as selective inhibitors [8]. In addition, Eg5 does not induce neuropathic side effects, which are found with microtubules inhibitors, due to the absence of the Eg5 protein in the adult peripheral nervous system [9, 10]. The literature review revealed that Eg5 inhibitors are divided into two groups, according to their mechanism of action within the Eg5 protein [11]. The first group is named ATP uncompetitive inhibitors, which targets site 1, α2/L5/α3 and the second group is known as ATP binding completive inhibitors, which targets site 2, helix-α4 and α6 pocket [12]. Monastrol (Fig. 1) is a ATP uncompetitive inhibitors, binds to site 1, that was discovered in 1999 by Mayer et al. through phenotypic screening methods and showed IC_50_ against Eg5 ATPase 30 uM [13]. However, the non-drug like properties and the weak activity of Monastrol encouraged the researchers to synthesize derivatives of Monastrol [14]. Lead optimisation of Monastrol through cyclization ester side-chain of the Monastrol into a cyclic ketone resulted in S-dimethylenastron (Fig. 1) [15]. The dimethylenastron showed an inhibitory activity of IC_50_ = 200 nM and a better fit within α2/L5/α3 binding pocket [16]. Further studies identified class II, dihydropyrimidine derivatives of Eg5 inhibitors include mon-97 and fluorastrol (Fig. 1) [11].

**Fig. 2:**
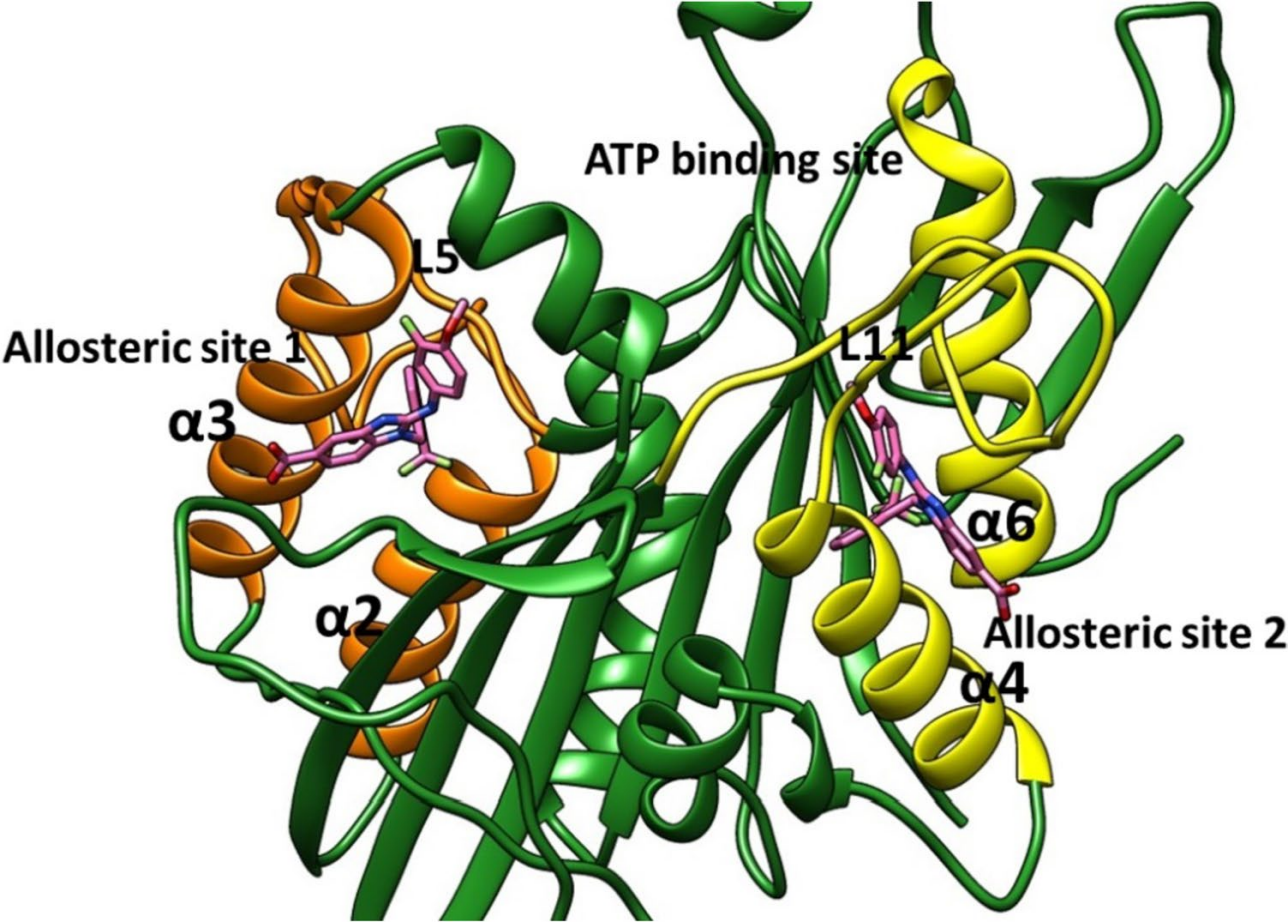
The binding pockets of Eg5 protein (PDB: 3ZCW) benzimidazole (pink, stick) binds to allosteric site (α2/L5/α3) Orange cartoon, allosteric site (α4/ α6/ L11) yellow cartoon

Ispinesib compound (SB-715992, CK0238273) was reported in 2002 by GlaxoSmithKline®, as an Eg5 inhibitor targets α2/L5/α3, which have a binding mode similar to Monastrol (Fig. 1) [17]. Binding of Ispinesib to the allosteric binding site, led to the motor function of the Eg5 protein at the ADPconformation locking and preventing the release of energy [18]. Ispinesib showed Eg5 ATPase IC_50_ less than 10 nM and illustrated a good safety profile [19]. However, the sixteen clinical trials were carried out on ispinesib, only fourteen were completed and two were stopped [20]. Unfortunately, none of these clinical trials led to deceive evidence about any benefits [20]. Lahue et al. in 2009 developed a series of substituted benzimidazoles, as Eg5 inhibitors target α2/L5/α3, and they found that benzimidazoles had promising Eg5 ATPase inhibitory activity [21]. However, the DMPK of selected compounds showed poor oral exposure in the rat [21]. Filanesib (Arry-520) was reported in 2009 by Array BioPharma®. Filanesib is a derivative of thiadiazole and it has Eg5 ATPase IC_50_ of 6 nM (Fig. 1) [22]. Around eight clinical trials were carried out for filanesib in patients with leukaemia and myeloma, and it showed most promising activity as an anticancer agent [20]. Despite the promising antitumor activity of these inhibitors, drug-resistant mutants located in L5 residues, called D130V and A133D, have been identified in the cell culture. These findings indicate the urgent need for developing a novel series of inhibitors that target the new allosteric pocket and use them either alone or with the reported Eg5 kinesin inhibitors, that target the α2/L5/α3 region [23]. Further extensive studies were performed by the researchers of GlaxoSmithKline® in 2006, they developed the biaryl derivatives (GSK-1 and GSK-2) as Eg5 inhibitors that target the new allosteric pocket formed of helix-α4 and helix-α6 (Fig. 2) [11]. The Ki value of GSK-1 and GSK-2 were 1.8 nM and 8.8 nM respectively against Eg5 protein. The most beneficial characteristic of GSK-1 and GSK-2 is that they were able to show potential inhibitory activity in ispinesib-resistant tumour cells, which carry D130V and A133D mutants of Eg5 [24]. However, GSK-1 and GSK-2 compounds were not clinically successful. Modified series of biphenyl compounds, such as PVZB1194, are under investigation like biphenyl Eg5 inhibitors [25]. These findings indicate the urgent need for developing a novel series of inhibitors that target the new allosteric pocket and use it either alone or with the reported Eg5 kinesin inhibitors that target the α2/L5/α3 region [23].

Biphenyl compounds were identified as Eg5 inhibitor and they showed potential antitumor activity in ispinesib-resistant tumour cells, that carry D130V and A133D mutations [26, 27]. Interestingly, it was found that benzimidazole derivatives bind to traditional allosteric pocket that consist of α2/L5/α3 and the second allosteric pocket α4/α6,L11 (Fig. 2). It exhibited potent anti-tumour activity in drug-resistant mutant cell lines [28, 29]. The benzimidazols bind to the α4/α6,L11 pocket, when Eg5 kinesin is in a complex with ADP or ATP, thus inhibit the release of ADP [27, 30]. In this study we aimed to identify a novel scaffold, as Eg5 inhibitors target allosteric pocket α4/α6/L11, using extensive computational studies, such as ligand-based drug design, molecular modelling, physicochemical and pharmacokinetics analyses, molecular dynamics simulations and binding free energy calculations. Furthermore, biological analyses were carried out to support the computational studies.

## Methods and materials

### Computational studies

#### Prepare the target protein

The co-crystal structure of mitotic kinesin Eg5 protein complexes with benzimidazole and ADP (PDB: 3ZCW) [27] were retrieved from PDB database https://www.rcsb.org/. The 3D structure of the protein was saved as PDB file format. The missing residues and loops (L11) were added using Modeller software [31].

#### Identify active pocket

The mitotic kinesin Eg5 contains the ADP/ATP binding pocket and several allosteric sites. The best-known one is the α2/L5/α3 allosteric site, which is around 10 Å from the ADP site. The other allosteric site is α4/α6/L11 allosteric, which is allocated 15 Å from the ADP site (Fig. 2). In this study we target the allosteric site α4/α6/L11, the L11 loop was built with modeller. In order to identify the allosteric pocket residues, the interactions between the benzimidazole and the allosteric pocket were investigated using Pymol and the key residues which within 4 Å form the centre of the reference ligand were identified.

#### Prepare the reference ligand

The co-crystalized ligand (2E)-2-(3-fluoranyl-4-methoxy-phenyl)imino-1-[[2(trifluoromethyl)phenyl] methyl]-3H-benzimidazole-5-carboxylic acid, which is bound to the Eg5 protein was used as a reference in this study. The ligand was prepared with AutoDock program [32]. The charges were assigned, the polar hydrogen atoms were added, the energy was minimized and the ligand was saved as pdbqt.

#### Ligand based virtual screening and physicochemical properties calculations

**Fig. 3:**
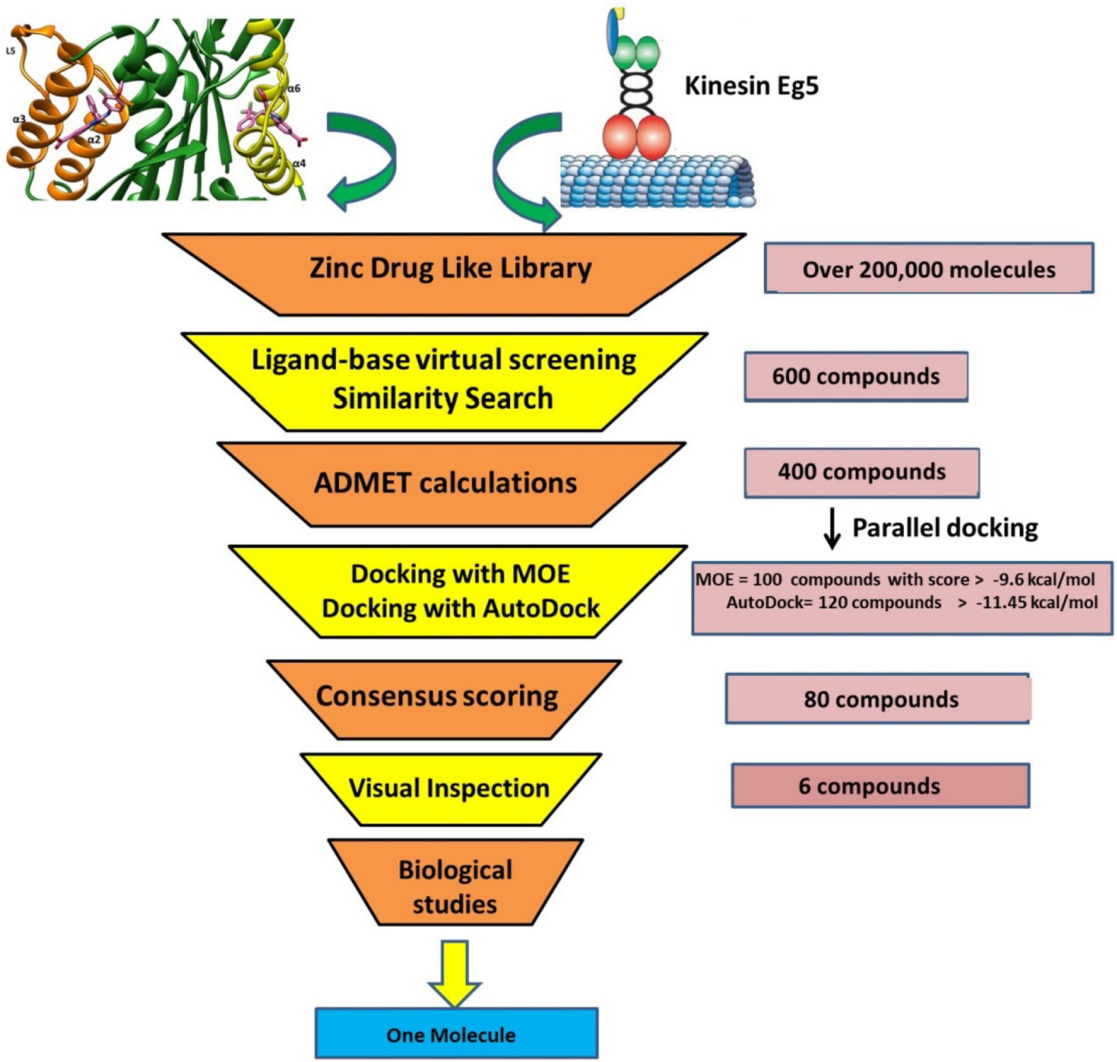
Schematic view of the ligand-based virtual screening

The virtual screening procedures consisted of ligand-based screening [33, 34], physicochemical properties filtration, molecular docking and visual inspection (Fig. 3). The reference ligand was used as template in a similarity search in SwissSimilarity [35]. The ZINC drug like library [36] (contains more than 200,000 molecules) was selected for the screening, and the electroshape method was applied as screening method. ElectroShape [37] method is a novel ligand-based virtual screening method, which combines electrostatic information and shape into a unified framework. The partial charge information was calculated and the chiral shape recognition (CSR) was incorporated. The CSR helps in distinguishing between the enantiomers. The method was validated using the Directory of useful Decoys (DUD), and showed a near doubling in enrichment ratio at 1%. During the similarity search, the compounds which showed score ≥ 0.8 were selected. After there were 600 molecules, which went through physicochemical properties or Lipinski**’**s rule filtration using DataWarrior software [38]. This step reduced the number of compounds to 400.

#### Molecular docking virtual screening

Molecular docking was carried out for the 400 compounds using two programs; AutoDock 4.2 [32] and MOE [39]. The validation of the applied protocol was performed, by re-docking the reference ligand in the binding site α4/α6/L11 of the Eg5 protein (PDB: 3ZCW).The RMSD values of Cα atoms were calculated with respect to the reference ligand. The RMSD values were found to be ≤ 1.0 Å between the X-ray structure and the best-scored conformations of the reference ligand. This is a good result and indicates the reliability of the docking protocol and the consensus of the methods. − 9.6 kcal/mol (reference ligand score calculated with MOE), while − 11.45 kcal /mol (he reference ligand score calculated with AutoDock).

#### Virtual screening with MOE

The ligands were prepared using MOE [39], the hydrogen atoms were added to the ligands and the energy was minimised until the gradient of energy with respect to the coordinates, fell below 0.05 kcal /mol/Å under the MMFF94X force field. The binding site was identified as the reference compound binding site. The Triangle Matcher method and the London ΔG scoring function were used in the docking protocol [40]. Refinement was carried out using the rescoring affinity ΔG method. The lowest energy pose was selected for each docked molecules yielding 100 compounds with a binding energy better than the reference compound, − 9.6 kcal mol^ − 1^.

#### Virtual screening with AutoDock 4.2

The reference compound was removed from the Eg5 crystal structure (PDB: 3ZCW, then AutoDock.4.2 converted the protein structure and the reference compound separately into pdbqt format. The polar hydrogen atoms were added and Kollman charges were assigned to the Eg5 protein while Gasteiger partial charges were assigned to the ligands. The non-polar hydrogen atoms were merged with their heavy atoms. The rotatable bonds in the ligands were defined using the AutoDock utility, AutoTors. The grid box was positioned at the centroid of the reference compound (X = − 3.454, Y = − 3.485 and Z = 9.098), the box size was 115 × 115 × 115 Å with a 0.27 A grid spacing. The grid map was calculated using Autogrid tool and saved as a gpf file. The molecular docking was performed using the Lamarckian genetic algorithm, each docking experiment was carried out a 100 times, and the configuration files were saved as dpf format. Raccoon software [41] was used to prepare all the 400 ligands to perform a molecular docking with AutoDock 4.2. The Raccoon software splits the multi-structure files of the molecules to separate PDBQT input files and generates configuration files for all the ligands. The results were sorted, according to the lowest predicted binding scores. Around 120 compounds showed predicted binding scores ≤  − 11.45 kcal mol ^ − 1^e reference ligand binding score was calculated with AutoDock).

#### Selection of compounds for biological testing

**Fig. 4:**
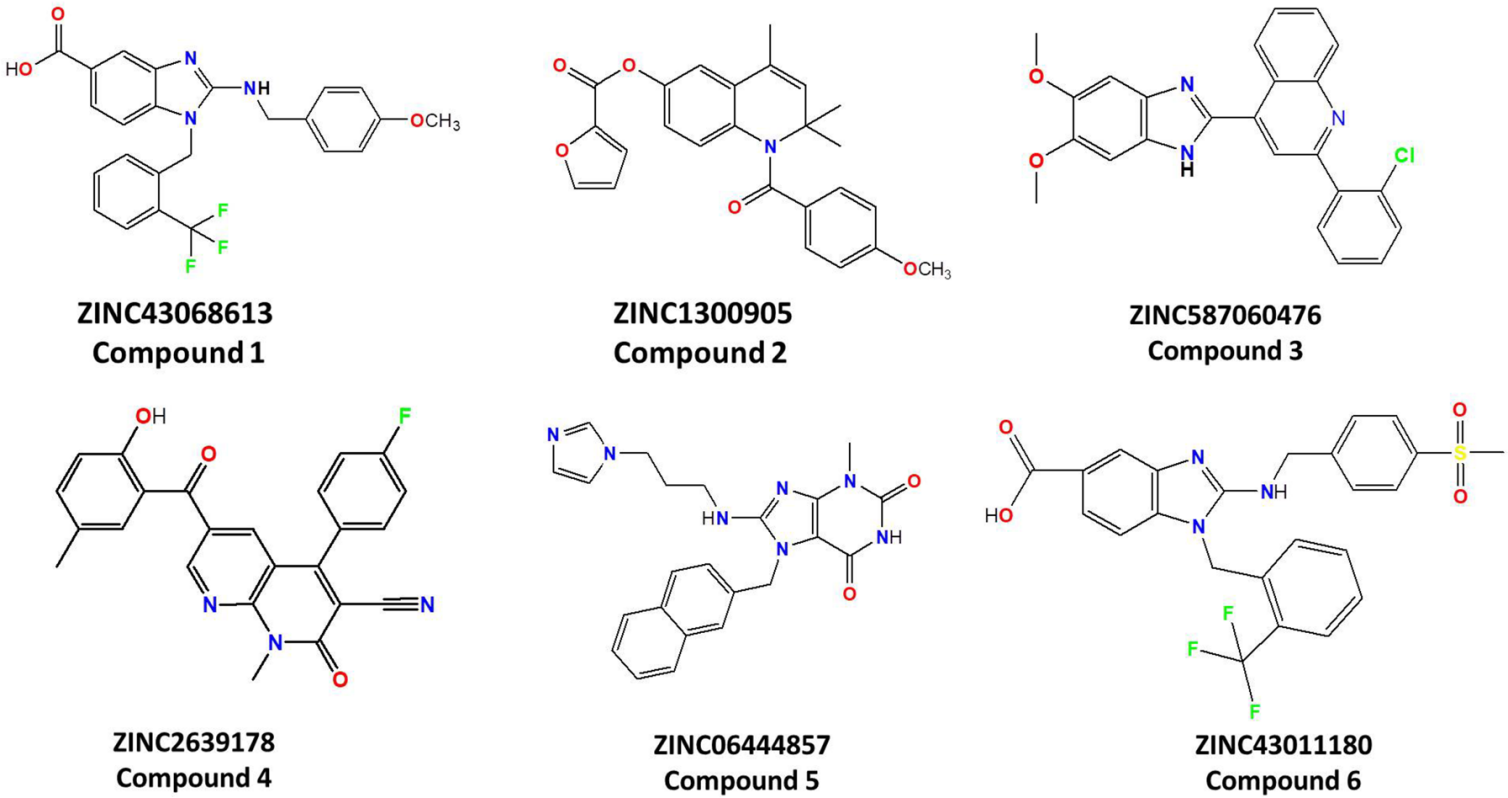
The chemical structure of the selected hits

The docked compounds which showed binding scores lower than the reference compound, in the two programs, were selected for visual inspection. This filtration step yielded 80 compounds. The visual inspection was performed considering these criteria; i) the molecules that showed good fitting within the allosteric pocket, ii) the molecules that formed at least one H-bond, iii) the molecules that formed hydrophobic interactions with the hydrophobic sub-pocket, that are lined with Leu288, Leu292, Leu293 and Leu332, iv) the molecules that shown aromatic interactions with the key residues Tyr104 and Tyr352. The visual inspections step gave a shortlist of 6 compounds (Fig. 4, Table 1). The shortlisted hits were screened for pan assay interference compounds (PAINS) using the online PAINS filters at https://www.cbligand.org/PAINS// The 6 compounds were purchased from https://vitasmlab.biz/ for experimental testing.

**Table 1:** ZINC ID and chemical names of shortlisted compounds and their binding scores by MOE, and AutoDock

ZINC ID	Cps Name	Chemical names	MOE score Kcalmol^-1^	AutoDock score kcal mol^ − 1^
Reference compound		1-(2-(trifluoromethyl)benzyl)-2-(3-fluoro-4-methoxyphenylamino)-2,3-dihydro-1H-benzo[d]imidazole-5-carboxylic acid	− 9.6	− 11.45
ZINC43068613	1	1-(2-(trifluoromethyl)benzyl)-2-(4-methoxybenzylamino)-1H-benzo[d]imidazole-5-carboxylic acid	− 10.1	− 11.90
ZINC1300905	2	1-(4-methoxybenzoyl)-2,2,4-trimethyl-1,2-dihydroquinolin-6-yl furan-2-carboxylate	− 9.9	− 12.20
ZINC587060476	3	2-(2-chlorophenyl)-4-(5,6-dimethoxy-1H-benzo[d]imidazol-2-yl)quinoline	− 10.2	− 13.42
ZINC2639178	4	4-(4-fluorophenyl)-6-(2-hydroxy-5-methylbenzoyl)-1-methyl-2-oxo-1,8-naphthyridine-3-carbonitrile	− 9.8	− 12.30
ZINC06444857	5	8-(3-(1H-imidazol-1-yl)propylamino)-3-methyl-7-((naphthalen-3-yl)methyl)-1H-purine-2,6(3H,7H)-dione	− 10.9	− 14.20
ZINC43011180	6	1-(2-(trifluoromethyl)benzyl)-2-(4-(methylsulfonyl)benzylamino)-1H-benzo[d]imidazole-5-carboxylic acid	− 10.3	− 13.90

#### Molecular dynamics (MD) simulations

The ligand coordinates of the docked complexes of Eg5-compound 5 and Eg5-reference ligand were used in MD simulations. The simulations were carried out 3 times for 200 ns for the reference compound, compound 5 complexes and Apo-protein using GROMACS 2022.4. [42] The Pdb2gmx tool was used to add hydrogen atoms to the Eg5 protein at PH 7. The topology file was generated using the Amber99-SB-ildnb force field [43]. The topology files of the reference compound and compound 5 were generated under the GAFF force field [44]. The ligand and protein complexes were centred in a chosen box with a minimum margin of 3.0 nm and filled with TIP3P water. The systems were neutralized by adding sodium and chloride ions to give an ionic strength of around 0.15 M. The energy minimization (5000 steps) was performed using the steepest descents. The simulation was conducted under the periodic boundary conditions and 300 K, as NPT only ensembles then [42]. The Particle Mesh Ewald (PME) method was applied for calculating long range electrostatics, and Van der Waals (VdW) interactions. The cut-off distance for the short-range VdW and Coulombic interactions was set to 1.2 nm [45]. The pressure was controlled by the Parrinello–Rahman barostat and temperature by the V-rescale thermostat. The simulation was integrated with a leap-frog algorithm over a 2 fs time step. Also, only H-bonds vibrations were constrained using the P-LINCS method. The molecular dynamics simulations were performed for 200 ns on BlueCrystal, the University of Bristol**’**s high-performance computing machine and the simulation analysis were performed using GROMACS tools. While Xmgrace [46] and gnuplot [47] were used for plotting the data, the molecular graphic manipulations and visualizations were performed using VMD [48], Chimera [49] and Pymol [50]. 

#### MM-GBSA binding free energy calculation

The binding free energy calculations of compound 5-protein complex and the reference complex were evaluated by the gmx_MMPBSA package with the GROMACS [51]. The snapshots were collected every 100 ps.

#### Principal component analysis (PCA)

PCA is one of the techniques that takes the trajectory of the molecular dynamics and extracts the main modes in the motion of the molecules [52]. The eigenvectors and eigenvalues were calculated along the first three Principal Components (PC) [53]. The amplitude of the eigenvector was identified by diagonalising the eigenvectors and eigenvalues matrix. The eigenvectors of the matrix gives the multidimensional space and the displacement of atoms in the protein along each direction [54]. In this study GROMACS tools were used to calculate the PCA.

### Biological studies

#### Enzyme inhibition assays

Microtubule (MT)-activated enzymatic Eg5-ATPase activity were measured in order to evaluate the inhibitory activity of the selected compounds at 20 µM. The MT—ATPase activity was analysed using Kinesin ELISA kit (BK060) (Cytoskeleton, Inc) and Monastrol was used as a control. The procedures were applied according to the manufacturer**’**s instructions [55]. The IC_50_ of compound 5 which were required for inhibiting 50% of Eg5 ATPase activity was measured using ELISA kit (BK060) (Cytoskeleton, Inc, Denver, CO) according to the manufacturer**’**s instructions [56]. 

#### Immunofluorescence assay

Hela cells (5 × 104/ well) were plated on coverslips in 6-well plates and treated with compound 5 and Monastrol at 20 µM for 24 h. Monastrol was used as a positive control, while 0.1% DMSO was used as a negative control. The cells were rinsed with PBS, fixed with 3.7% paraformaldehyde, and permeabilized with 0.1% Triton X-100. The cells were blocked with 1% BSA in PBS for 1 h prior to incubation with anti-β-tubulin mouse monoclonal antibody (#86,298, Cell Signaling, San Francisco, CA, USA) overnight at 4 °C. The cells were washed with PBS for 1 h in the dark, and then incubated with Alexa Fluor® 488 secondary antibodies (Abcam). The cellular microtubules were observed with a fluorescence microscope (Olympus BX43, Japan).

## Results and discussion

### Virtual screening (VS)

The virtual screening procedure was described in great details in methods and Fig. 3. In summary, the ligand-based virtual screening relies on the structure of the reference ligand of Eg5 protein (PDB: 3ZCW). A library of drug-like ZINC database was screened to obtain lead compounds. Subsequently, 600 compounds were obtained, then the physicochemical properties and molecular docking studies were carried out and finally 80 compounds were selected. Further, visual inspections were applied and based on the binding mode within the target pocket, the binding score, the H-bond, the hydrophobic and aromatic interactions of the potential compounds with an allosteric pocket of Eg5 protein. Six compounds, named ZINC43068613 (compound 1), ZINC1300905 (compound 2), ZINC587060476 (compound 3), ZINC2639178 (compound 4), ZINC06444857 (compound 5) and ZINC43011180 (compound 6), were selected for further analysis (Fig. 4, Table 1). Interestingly, compound 1 and 6 were identified by Lahue [21], as Eg5 inhibitors that target α2/L5/α3 pocket, however in our study both compounds were identified as α4/α6/L11 inhibitors.

### Molecular docking

**Fig. 5:**
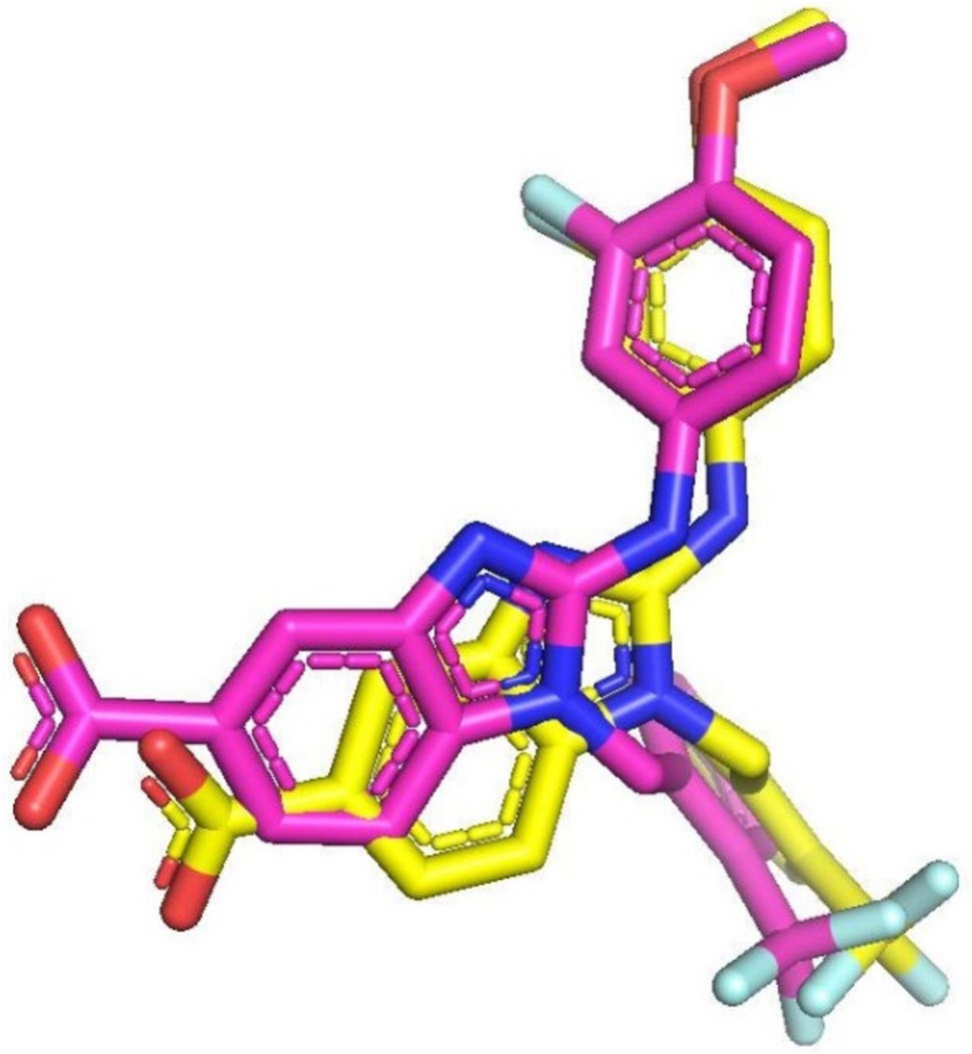
Superimposition of the original co-crystalized ligand benzimidazole (pink) and re-docked pose (yellow) in Eg5 allosteric site

**Fig. 6:**
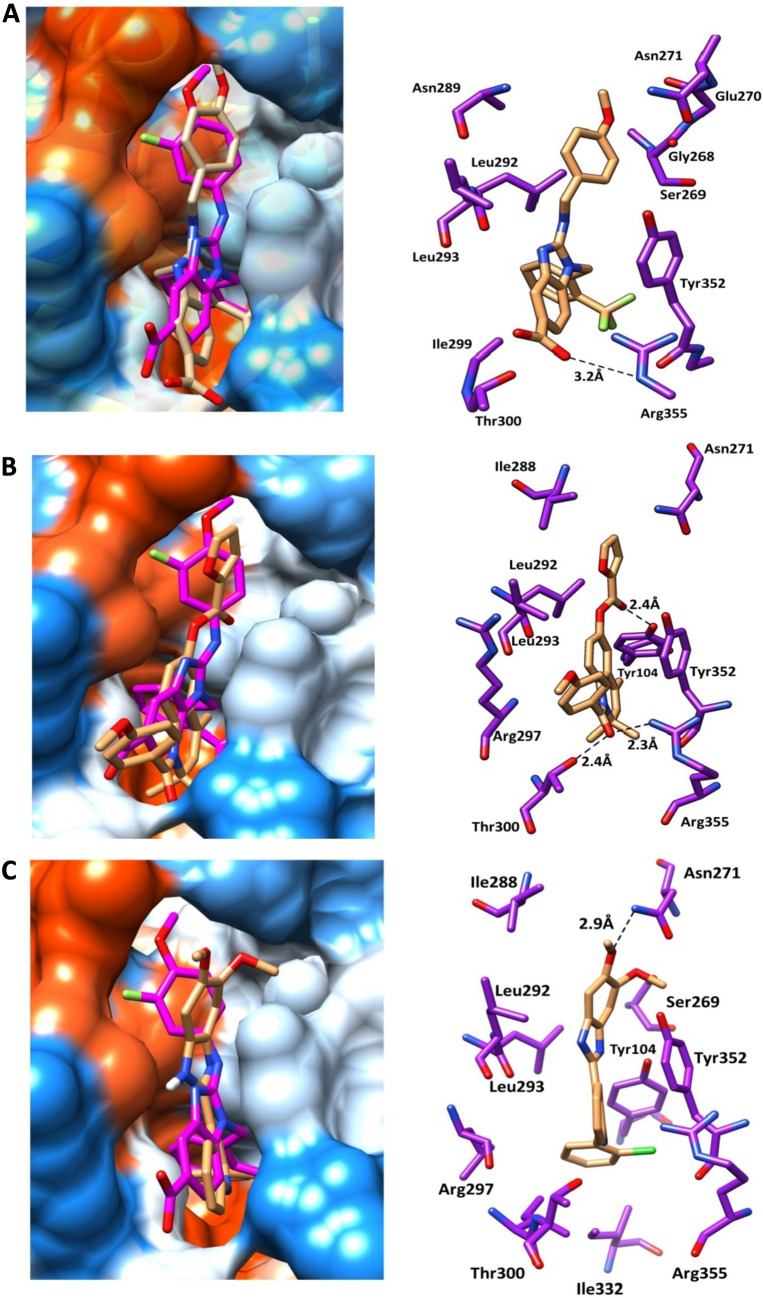

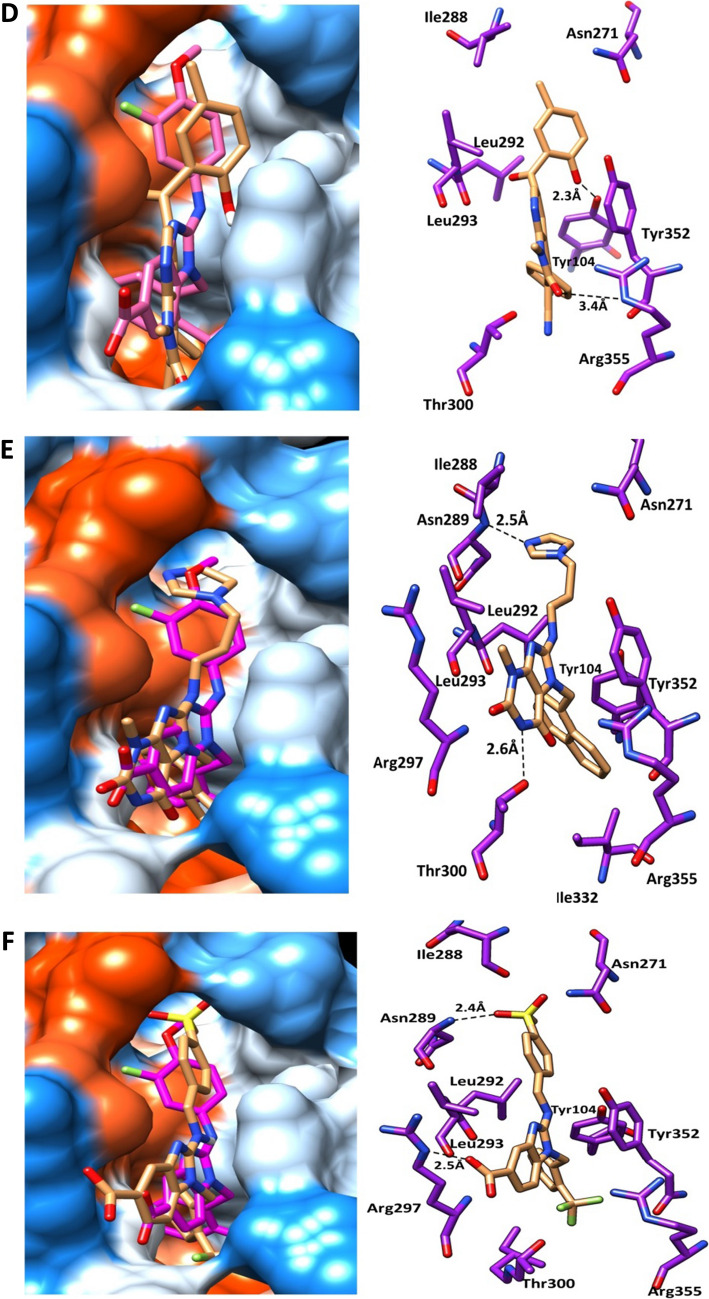
Binding mode **A** compound 1, **B** compound 2, **C** compound 3. Right Panel represented the 3D interactions of compounds and the pocket**’**s residues (purple, stick). H-bond represented as black dotted-line.Left panel represented the selected compounds (brown, stick) compared to the reference compound (magenta, stick) within the binding surface (Orange; hydrophobic, white; neutral; blue; hydrophilic). Binding mode **D** compound 4, **E** compound 5, **F** compound 6. Right Panel represented the 3D interactions of compounds and the pocket**’**s residues (purple, stick). H-bond represented as black dotted-line. Left panel represented the selected compounds (brown, stick) compared to the reference compound (magenta, stick) within the binding surface (Orange; hydrophobic, white; neutral; blue; hydrophilic)

The re-docked reference ligand showed a similar binding mode to co-crystalized ligand (Fig. 5), which suggests the docking protocol is efficient and solid. The docking results are summarized in Table 2, and Fig. 6. We can conclude that the shortlisted compounds are mimicking the same binding mode as the reference ligand of Eg5 protein. All the ligands occupied the back hydrophobic sub-pocket, that formed of Ile288. In addition, all molecules showed hydrophobic interactions with the key residues Leu292, Leu293 and Ile299. Furthermore, the key residues Tyr104 and Tyr352 formed pi-pi interactions with the six selected hits. All the six compounds formed H-bonds with the allosteric pocket residues. In further detail, compound 1 and compound 2 formed hydrogen bonds with the key residue Arg355. The C=O of compound 1 formed H-bond with NH-Arg355, while the C=O group of compound 2 formed two H-bonds with OH-Thr300 and NH-Arg355 at distances of 2.4 Å and 2.3 Å respectively. An additional H-bond was observed between compound 2 and Tyr352. Both compounds were embedded in the front and back hydrophobic pockets (Fig. 6). Interestingly compound 1 has a similar structure to the reference ligand, while the fluoro atom in position 3 of methoxybenzyl-amine group of the reference compound showed closer contact to Leu292 and Leu293 than compound 1. The methoxy group of compound 3 and compound 4 formed H-bonds with the allosteric pocket. Methoxy group of compound 3 formed an H-bond with NH-Asn271 at distance 2.9 Å, while the methoxy group of compound 4 formed an H-bond with OH-Tyr104 at a distance of 2.9 Å. Compound 4 formed another H-bond with NH-Arg355 at a distance of 3.4 Å. Both compounds 5 and 6 formed two H-bonds, compound 5 formed an H-bond between N-imidazole and NH-Asn289 at a distance of 2.5 Å and the second H-bond formed between N of purine and OH-Thr300 at a distance of 2.6 Å. While compound 6 formed an H-bond between the oxygen atom of SO_2_ and NH-Asn289 at a distance of 2.4 Å and the second H-bond formed between OH and NH-Arg297 at a distance of 2.5 Å. Both compounds showed hydrophobic interactions with the residues Leu292, Leu293 and Leu299.

**Table 2:** Docking results and interactions of hits compound

ZINC ID	Cps NO	Residues of proteins	Moieties of compounds	Type of interactions
ZINC43068613	1	NH-Arg355 Val278,Leu292,Leu293 Tyr104 Tyr352	C=O Benzyl group trifluromethylbenzyl Benzimidazole	H-bond (3.2 Å) Hydrophobic π – π π – π
ZINC1300905	2	OH-Tyr104 OH-Thr300 NH-Arg355 Tyr104,Tyr352 Ile288 Ile299 Leu292, Leu293	C=O C=O C=O Quinoline Furan Methoxybenzoyl Quinoline	H-bond(2.4 Å) H-bond(2.4 Å) H-bond(2.3 Å) π–π Hydrophobic Hydrophobic Hydrophobic
ZINC587060476	3	OCH_3_ Tyr104 Tyr352 Arg355 Leu292,Leu293 Leu266,Leu295,Ile332	NH-Asn271 Chloro-phenyl Benzimidazole Quinolone Benzimidazole Chloro phenyl	H-bond(2.9 Å) ππ π–π Arene-cation Hydrophobic Hydrophobic
ZINC2639178	4	OCH_3_ C=O Tyr104 Tyr352 Ile288 Leu292,Leu293 Ile299,Ile332,Ala356	OH-Tyr104 NH-Arg355 Fluro-phenyl naphthyridine Methyl-benzoyl naphthyridine Flurophenyl	H-bond(2.3 Å) H-bond(3.4 Å) π–π π–π Hydrophobic Hydrophobic Hydrophobic
ZINC06444857	5	NH-Asn289 OH-Thr300 Tyr104,Tyr352 Ile288 Leu292,Leu293,Ile332	N-imidazole N-Purine Naphthalene Imidazole Naphthalene	H-bond(2.5 Å) H-bond(2.6 Å) π–π Hydrophobic Hydrophobic
ZINC43011180	6	S=O OH Tyr104 Tyr352 Ile288,Leu292,Leu293,Ile299	NH-Asn289 NH-Arg297 Phenyl group Benzimidazole Benzyl and phenyl	H-bond(2.4 Å) H-bond(2.5 Å) π–π π–π Hydrophobic

### Physiochemical and ADME properties

The predicted physicochemical properties of the selected hits, were calculated using DataWarrior and the Lipinski rule of five [57]. The co-crystalized ligand was used as a reference in these calculations, the results were summarised in Table 3. The values of physicochemical parameters of the 6 hits are H-bond donors ≤ 5, the H-bond acceptors ≤ 10, logP_w/o_ ≤ 5, the rotatable bond ≤ 10, total polar surface area is ≤ 140 A [2], and the molecular weights ≤ 500 g/mol. The results indicate that the selected hits follow Lipinski rule, expect compound 6 as it showed a molecular weight of 503.49 g/mol which is slightly more than the Lipinski limit, but it is still in the accepted range. The log S of the 6 hits were calculated and compounds (1, 2, 4, 5, and 6) showed moderate solubility. However, compound 3 showed slightly poor solubility, but so did the reference compound. These results revealed that 5 hits are exhibiting drug-like properties and are absorbed well by the biological system. The pharmacokinetic (absorption, distribution, metabolism, excretion) properties of the six hits and reference compound were investigated (Table 4). The first five compounds showed high absorption from the intestine similar to the reference compound, while compound 6 showed low absorption. All the hits do not penetrate the blood brain barrier. Cytochrome P450 is one of the important detoxification enzymes in humans; many drugs are activated or inhibited by cytochrome P450. It was found that compounds 1 and 3 are predicted to be deactivated by CYP2D6 similar to the reference compound. Compounds 2, 4, 5, and 6 are predicted to be non-inhibitors for CYP2D6. Regarding excretion compounds 1, 3, 4, and 6 are not substrates to renal OCT2 enzyme, like the reference compound, while compound 2 and 5 are substrates to the renal OCT2 enzyme.

**Table 3:** Predicted the physicochemical properties of the selected compounds

ZINC ID	Cps NO	M.wt g/mol	No rotatable bonds	No H-bond acceptors	No H-bond donors	Log P_o/w_	Log S	TPSA A^2^
Reference compound		461.41	7	7	3	5.3	− 6.25 (Poor)	73.83
ZINC43068613	1	455.43	7	5	2	5.0	− 5.90 (Moderate)	76.38
ZINC1300905	2	417.45	6	5	0	3.97	− 5.33 (Moderate)	68.98
ZINC587060476	3	415.87	4	4	1	2.87	− 6.33 (Poor)	60.03
ZINC2639178	4	413.40	3	6	1	2.76	− 5.13 (Moderate)	95.98
ZINC06444857	5	429.47	7	4	2	2.51	− 5.37 (Moderate)	102.53
ZINC43011180	6	503.49	8	8	2	2.32	− 5.71 (Moderate)	109.67

**Table 4:** ADME (Absorption, Distribution, Metabolism, and Excretion) properties of selected compounds

ZINC ID	Cps No	Absorption	Distribution BBB Permeability	Metabolism CYP2D6 prediction	Renal OCT2 substrate
Reference compound		High	NO	Yes	NO
ZINC43068613	1	High	NO	Yes	NO
ZINC1300905	2	High	NO	Non-inhibitor	Yes
ZINC587060476	3	High	NO	Yes	NO
ZINC2639178	4	High	NO	Non-inhibitor	NO
ZINC06444857	5	High	NO	Non-inhibitor	Yes
ZINC43011180	6	Low	NO	Non-inhibitor	NO

### Toxicity studies

The toxicity prediction study of the hits and reference compound are summarised in Table 5, the hits were expected to have a non-toxicity profile. The hits and the reference compound are predicted to be non–mutagenic. In addition, the hits were predicted to be non-carcinogenic based on the FDA rodent carcinogenicity model. The oral rate acute toxicity LD_50_ values of all the compounds, except compound 4, are around 2.42–2.78 mol/kg. This is similar to the reference compound**’**s rate of 2.50 mol/kg, while the LD_50_ of compound 4 is 3.09 mol/kg. The six compounds showed rat chronic lowest observed adverse effect level (LOAEL) values that ranged from − 0.02 to 1.51 g/kg, which is extremely close to the reference compound 1.10 g/kg. Interestingly, the hits are non-irritant to the skin.

**Table 5:** Predicted toxicity of selected hits

ZINC ID	Cps No	Mutagenic	Oral rat acute toxicity (LD50) (mol/kg)	Oral rat chronic toxicity (LOAEL)	Skin irritation
Reference compound		None	2.50	1.10	NO
ZINC43068613	1	None	2.47	1.25	NO
ZINC1300905	2	None	2.78	1.51	NO
ZINC587060476	3	None	2.42	0.38	NO
ZINC2639178	4	None	3.09	1.01	NO
ZINC06444857	5	None	2.48	− 0.02	NO
ZINC43011180	6	Llow	2.47	1.46	NO

### Biological studies of selected hits

#### MT ATPase assay

**Fig. 7:**
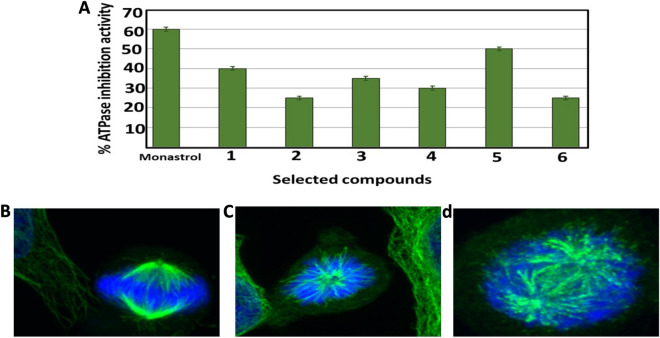
**A** Represented enzymatic Eg5 –ATPase inhibition activity of the 6 hits. Each result is a mean of 3 replicate samples and values are represented as % inhibition (± standard deviation). Immunofluorescence assay of monastrol and compound 5, the Hela cells were treated for 24 h with **B** DMSO as a negative control, **C** Monastrol as a positive control, **D** compound 5 at 10 µM then fixed and stained with anti-α-tubulin antibody (green) and with DAPI for DNA (blue) to visualize the microtubules

The Eg5 ATPase activity of the six hits at 10 µM was investigated in comparison to Monastrol (10 µM), as it acted as a positive control compound. The results are shown in Fig. 7A and indicate that compound 5 showed significant anti MT ATPase activity. Compound 5 showed 50% inhibition activity, in comparison to Monastrol with 60% inhibition activity. Similarly compounds 1, 3 and 4 caused 40, 35 and 30% inhibition activity respectively. Compounds 2 and 6 showed the lowest inhibitory activity at around 25%. The results revealed that compound 5 showed the most promising inhibition activity, which prompted the measurement of its IC_50_ against Eg5 ATPase; it was found that the IC_50_ of compound 5 is 2.37 ± 0.15 µM. Although some compounds showed better IC_50_ than compound 5, however cellular resistance was developed for these compounds, which makes compound 5 a promising lead compound with new scaffolding.

#### Immunofluorescence assay and inhibition of mitotic spindle formation

Further investigation of compound 5 was performed, which consisted of an immunofluorescence assay that was used to analyse the mechanism of compound 5 on the organisation of the microtubules into mitotic spindles during divisions of the cells. The cells were treated with the negative control (10 µM), which showed typical bipolar spindles (Fig. 7B), while Monastrol and compound 5 (10 µM) treated cells formed monopolar spindle profiles (Fig. 7C, D). These results revealed that compound 5 caused tubulin assembly distortion with irregular morphology, showing a typical mitotic arrest similar to Monastrol.

### Molecular dynamic simulations

Molecular dynamics (MD) provide deep insights into ligand-receptor interactions and a wide understanding of the ligand-receptor conformational change over the time. MD simulations consider the flexibility of the protein, which is not taken into account by molecular docking programs, which results in more reliable results. In this study molecular dynamics were performed 3 times for 200 ns with different initial velocities. The top docked poses of compound 5 and reference compound complexes with the allosteric site of Eg5 protein were selected, and the Apo-protein for molecular dynamic simulations using GROMACS software.

#### Root mean square deviations (RMSDs) and root mean square fluctuations (RMSF)

**Fig. 8:**
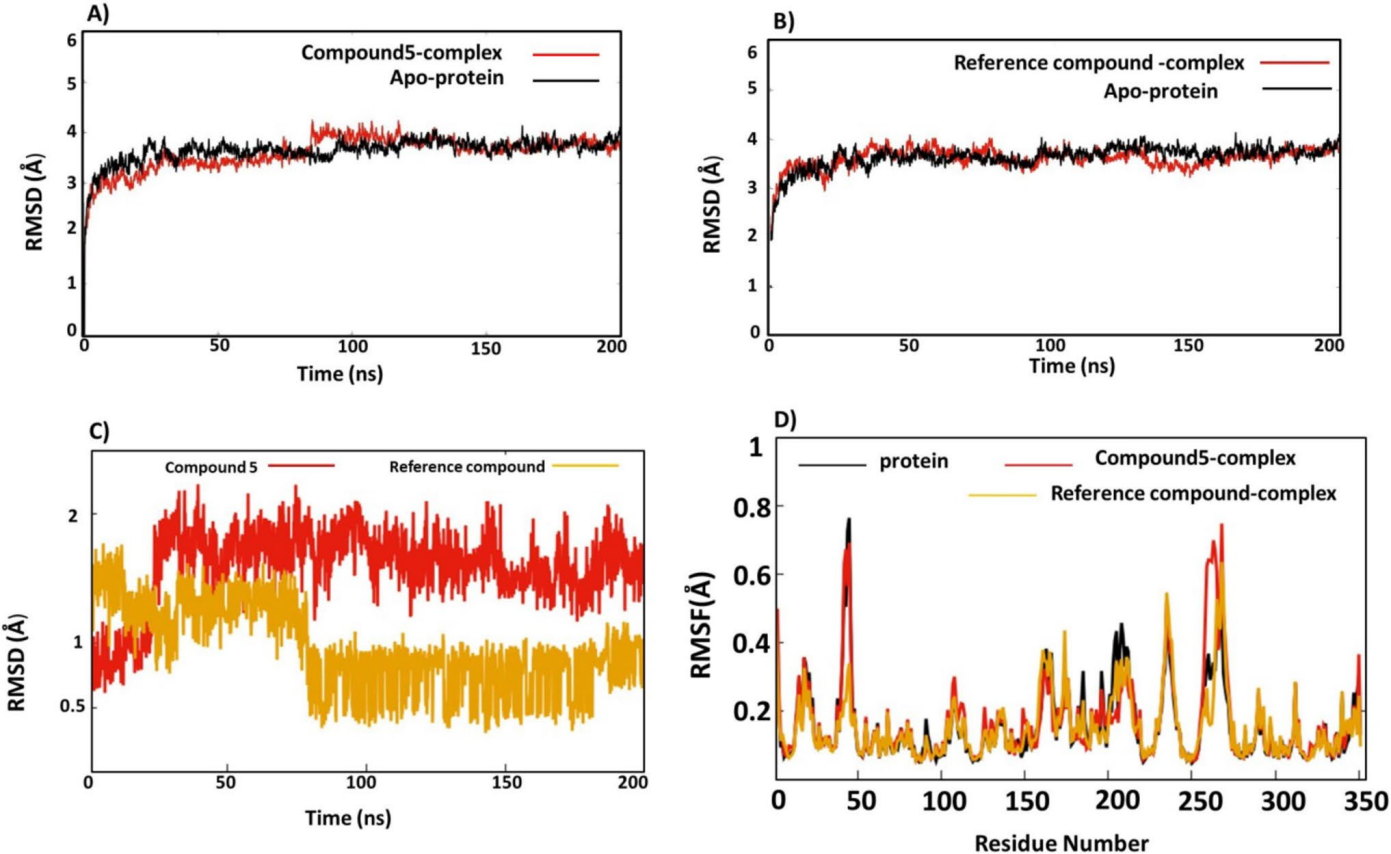
**A** RMSD of Cα of compound 5-complex (red) and Cα of Apo-protein (black). **B** RMSD of Cα of native ligand complex (red) and Apo-protein (black). **C** RMSD of compound 5 (red), native ligand (yellow). **D** RMSF of compound 5-complex (red), native ligand –complex (yellow) and Apo-protein (black)

In order to investigate the stability of compound 5—Eg5 complex and analyse any conformational changes, the Cα root-mean-square deviations (RMSDs) of inhibitor complex was calculated and compared with the reference ligand complex and Apo-protein. The average values of RMSD of the complexes are represented in Table S1. The average RMSD of the Apo-protein, compound 5-complex and reference compound—complex showed values 3.5 ± 0.2 Å, 3.0 ± 0.3 Å and 3.1 ± 0.3 Å. It could be observed from Fig. 12A, B that the compound 5 complex reached equilibrium at 20 ns, with an average RMSD value around 3 Å, the complex continued smoothly until the end of the simulation. Compound 5 remained in a similar position to the original pose in runs 1, 2 and 3. Interestingly, RMSD of compound 5-complex is similar to RMSD of Apo-protein during most of the simulations, the RMSD plot of compound 5 complex and Apo-protein overlap in most of the simulations (Fig. 8A). The RMSD of the reference compound complex reached equilibrium at 20 ns with a value around 3 Å. RMSD plot continued smoothly and no significant changes occurred during the remaining time period of the trajectory (Fig. 8B). These results indicate the stability of the compound 5 complex and its similarity to the reference ligand complex and Apo-protein.

In order to understand the binding mode of compound 5 and its stability within the allosteric pocket, the RMSD of the compound 5 alone was calculated relative to the starting docked pose, and compared with the RMSD of the reference compound (Fig. 8C). It could be seen that RMSD of compound 5 reached equilibrium at 20 ns and the plot continued in a straightforward manner until the end. The average RMSD value was 2 Å. On the other hand, the reference compound reached the equilibrium at 10 ns with RMSD value 1.5 Å, at 70 ns the RMSD decreased sharply to around 0.5 Å and continued like that until the end of the 200 ns. These results reveal compound 5 adopted one stable conformation over the dynamics. We investigated the conformations of the reference ligand at RMSD 1.5 Å and 0.5 Å. It was found that the fluoro-methoxy phenyl group adopted two conformations at 1.5 Å while at 0.5 Å; the fluoro-methxy group adopted the same conformation (Figure S1). The overall results are that compound 5-complex is stable over the dynamics and adopts one conformation during the entire trajectory.

Root-mean-square fluctuations (RMSF) of Cα atoms allow direct insight into the structural fluctuations and the degree of the residues flexibility in the protein. RMSF values of Cα atoms of compound 5-complex, reference compound-complex and Apo-protein were computed (Fig. 8D). The average RMSF (Table S1) of the Apo-protein, compound 5-complex and reference compound were 0.33 ± 0.4 Å, 0.28 ± 0.30 Å and 0.30 ± 0.3 Å. A close observation of RMSF plot reveals that the residues of the three systems displayed a low degree of flexibility with an average value 0.3 Å. The highest two peaks with RMSF were around 0.8 Å, the first one is between Asp43-Glu48 which is a loop, and the second one is between residues Val264-Asn271, which represents another loop. The three systems show nearly the same RMSF profile with low fluctuations in allosteric site α4 (292–297) and α6 (348–355). Overall, the three systems show lower residue fluctuations and lower RMSF values, than the Apo-protein residues. Most of the fluctuations are in the loops. These small ranges of RMSFs demonstrate that compound 5 is capable of forming suitable and stable interactions with the allosteric pocket during the dynamics. These results are in accordance with the findings from the RMSD results.

#### Solvent accessible surface area (SASA) and radius of gyration (Rg) analyses

**Fig. 9:**
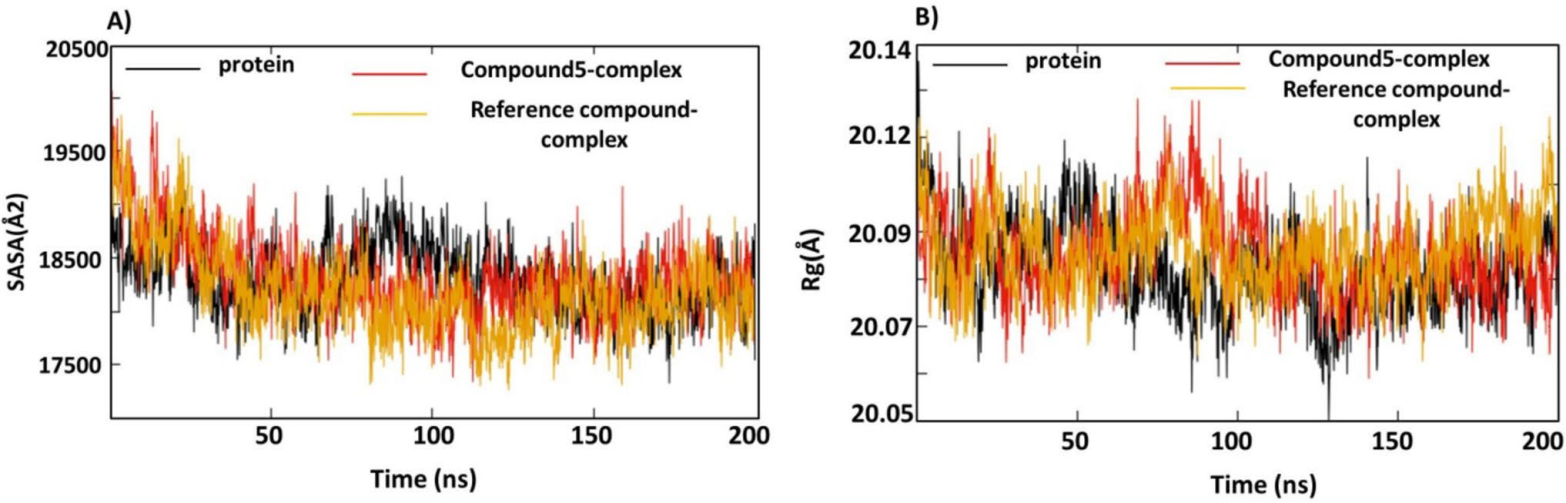
**A** SASA of compound 5-complex (red), native ligand –complex (orange) and Apo protein (black). **B** Rg of compound 5-complex (red), native ligand –complex (orange) and Apo protein (black)

SASA is the surface area accessible to the water molecule; SASA calculations can help to investigate the conformational dynamics of the compound 5—Eg5 protein. The average SASA values (Fig. 9A) for compound 5—protein, reference compound—complex and the Apo—proteins are 18,200Å^2^, 18,200Å^2^ and 18,300Å^2^. It could be seen that the SASA plots for compound 5 and reference compound complexes have overlapped in all the simulations. The SASA for both complexes started high with value 19,500, then reduced gradually until 50 ns to be 18,200 Å^2^ and continued smoothly until the end of trajectories. The SASA of Apo-protein started with 18,500 Å^2^ then reduced to 18,300 Å^2^, suddenly at 80 ns the SASA value of Apo-protein increased again to 19,000 Å^2^ until 110 ns and decreased again to 18,300Å^2^. The SASA results reveal the stability of compound 5—complex is similar to the reference compound, and that there were less inner residues interacting with the surroundings. The SASA fluctuations of the Apo-protein are slightly higher than the compound 5 complex which indicates that there are more inner residues in contact with the solvent, and binding compound 5 to the allosteric site reduced this contact and stabilized the Eg5 protein.

Radius of gyration (Rg) is another parameter which is linked with the tertiary structure and the general conformational state defining our understanding of compactness and the folding of proteins. The Rg of the three complexes were calculated and the results are represented in Fig. 9B. The three systems showed the same Rg average 20.09 Å. A fluctuation of 0.1 Å, around the average values were observed for compound 5 and reference complexes (Fig. 9B) at 70 ns and immediately after 20 ns the graph returned to its average value. These sudden deviations may be attributed to protein**’**s packing. All three graphs attained equilibrium around their average value, thus suggesting that the complex relaxed throughout the dynamics and confirms that compound 5-complex remained compact, and the folding of the Eg5 protein was maintained.

#### H-bonds and ligand–protein contact

**Fig. 10:**
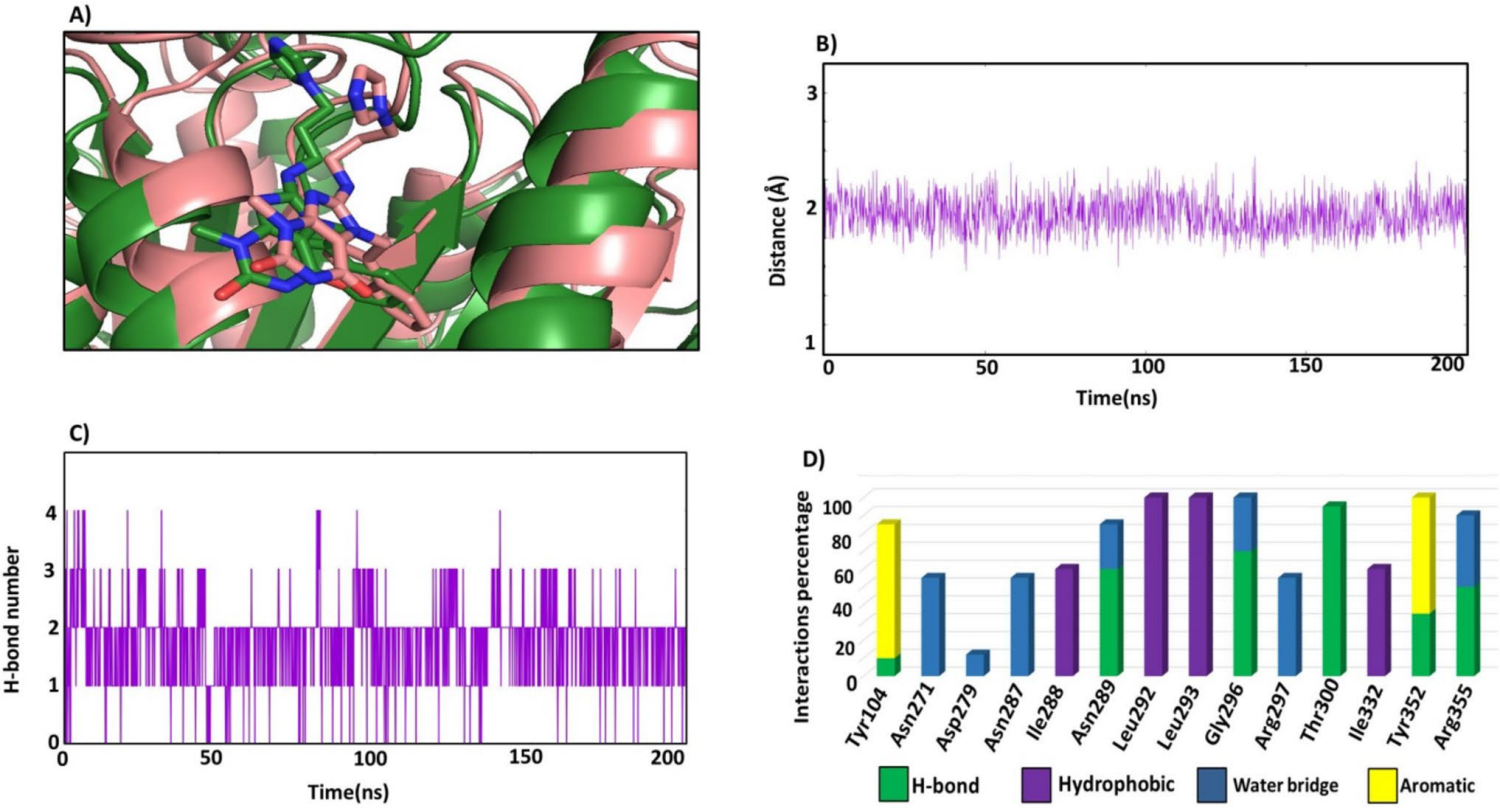
**A** Snapshot of binding mode of compound 5 at the initial of the simulation (green) and at the end of 200 ns. **B** The Distance from the centre of mass of Eg5 allosteric site to the centre of mass of the compound 5. **C** No of H-bonds of compound 5 with the allosteric pocket**’**s residues. **D** Protein–ligand contact mapping for Eg5 protein with compound 5

The MD trajectory of compound 5 was analysed to gain more information about the stability of the ligand within the binding pocket and intermolecular interactions (H-bonds, hydrophobic, pi-pi interactions) over the simulation. It was found that compound 5 occupied the same position within the binding pocket from the starting point till the end (Fig. 10A). The distance from the centre of mass of compound 5 to the centre of mass of the allosteric pocket was around 2 Å (Fig. 10B) and the plot was smooth and stable over the 200 ns, these results confirm the stability of the inhibitor within the pocket. Investigations about the intermolecular interactions of compound 5 over the dynamics showed that compound 5 formed four H-bonds with the binding site (Fig. 10C) with the interactions percentage being 97%. The most stable and continuous H-bond was with Thr300 which persists over the simulation with occupancy of 95%. Furthermore, at 20 ns two H-bonds formed between compound 5 and residues Gly296 and Arg355 with percentages of 70% and 50% respectively, but when Arg355 or Gly296 moved away from compound 5, these two H-bonds were broken. At the same time, another two H-bonds formed between compound 5 and residue Asn289 and Tyr352 (Fig. 10D). It could be observed compound 5 was able to maintain the initial binding mode of the simulation through forming strong polar interactions with residues Asn271, Asp279, Asn287 and Arg297. Besides the pi-pi interactions with Tyr104 and Tyr352 (Fig. 10D). Forming continuous H-bonds with Asn289, Gly296, Tyr352 and Arg355 with the average percentage more than 50%, this suggests that the H-bonds may be the main reason for the stabilization of compound 5. In particular, the stable H-bond formed with Thr300 was almost preserved in throughout the entire dynamics. Additional hydrophobic interactions were established with Ile288, Leu292, Leu293, and Ile332, as they had high occupancy which was greater than 60% (Fig. 10D) so fortified the stability of the ligand over the whole trajectory.

### Principle component analysis (PCA)

**Fig. 11:**
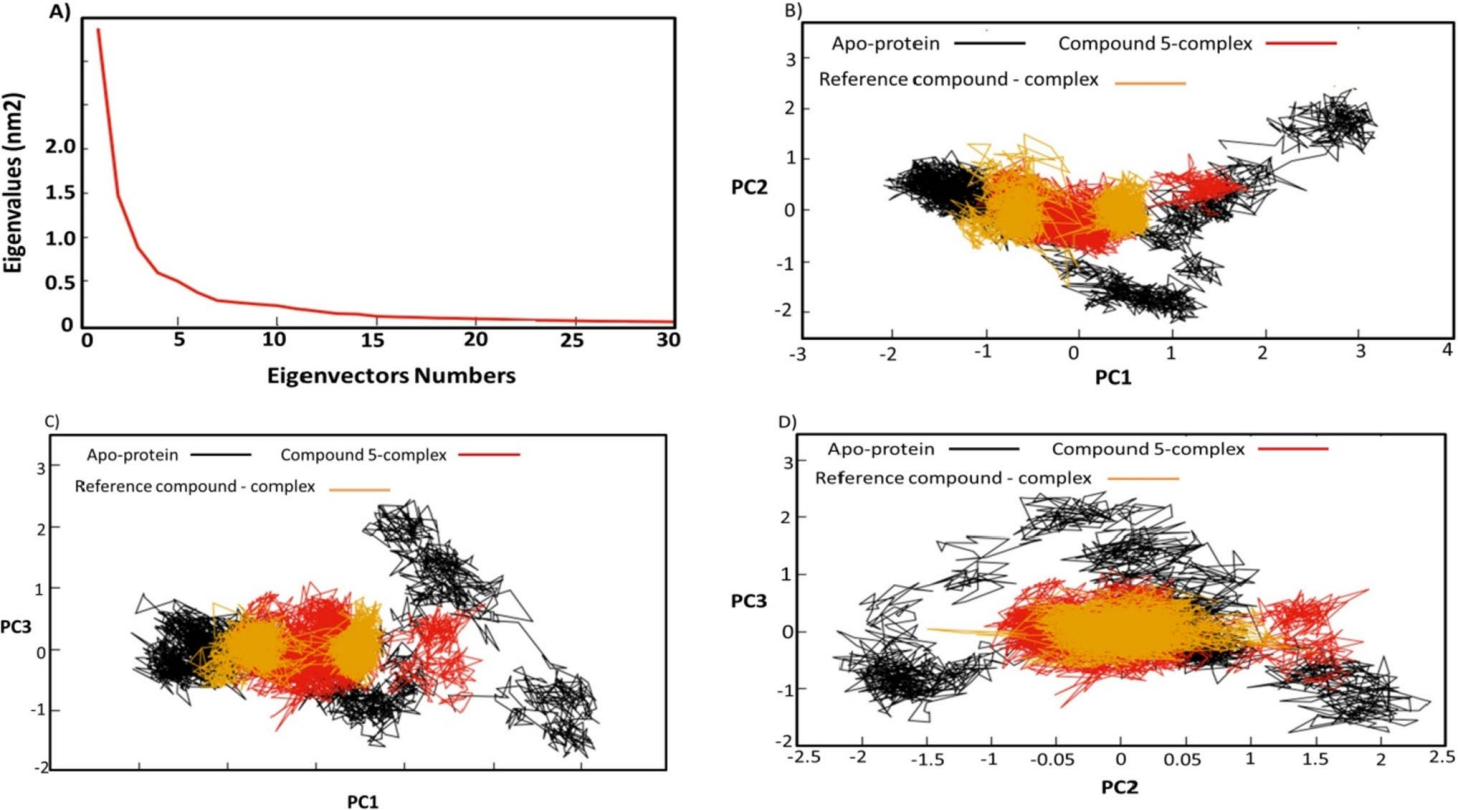
**A** The changes in the eigenvalues with increasing the eigenvectors. **B** The projection of each trajectory on the first two eigenvectors. **C** The projection of each trajectory on the first and third eigenvectors. **D** The projection of each trajectory on the second and third eigenvectors

The collective motion of compound 5—Eg5 protein, reference compound—Eg5 protein and Apo-protein was computed from the trajectories using PCA method. The method based on the constructions of the diagonal covariance matrix from Cα atoms of the Eg5 protein, which captures the global motion of the atoms through eigenvectors or the principal components (PCs) and eigenvalues. The eigenvectors explain the global direction of motion of the atoms, while the eigenvalues represent the atomic contribution of motion in MD trajectories of each system. The reduction in the subspace size was determined using the scree plot, the distribution of the eigenvectors versus eigenvalues. Figure 11A demonstrates a sharp fall in the slope at the fifth PC. The first eigenvector accounted for 78.9% of the overall variance, the first three eigenvectors together accounted for roughly 92% of the total variance. The first three eigenvectors were selected to calculate the reduced subspace.

### Bi-dimensional projection studies

The projecting of each trajectory of the three systems was represented in Figs. 11B–D. The conformational subspace of the systems was evaluated using the first three eigenvectors of the total Cα. The PCA study can help in understanding the dynamic behaviour of compound 5—complex and compare that with dynamics behaviour of the reference complex and Apo-protein. The PCA of the first and second eigenvectors revealed that the conformational clusters of compound 5-complex are well defined and covered a minimal number of subspaces (Figs. 11B) in comparison to the Apo-protein which occupied large subspaces. Moreover, the trajectories of compound 5 and the reference compound overlapped in most of the dynamics with different average structures. Similarly, the PC between the eigenvectors one and three (Fig. 11C) and between eigenvectors two and three (Fig. 11D) showed nearly the same atomic motions. These results indicate that compound 5 and reference compound complexes cover the minimum number of subspace, while the Apo-protein showed large atomic motions and conformational changes. The trajectories of compound 5—Eg5 and reference compound—Eg5 overlapped in most of the PCA analyses and occupied a small subspace range. These finding indicate that compound 5 showed restricted subspaces in a complex with Eg5 protein, leading to a well-defined internal motion behaviour, which stabilized the complex better than Apo-protein.

### MM/GBSA binding free energy

The MM/GBSA method was used to calculate the free binding energy of compound 5 and the reference compound. The components of the binding free energy were represented in Table 6. Compound 5 has a binding energy slightly higher than the reference. The free binding energy for compound 5—complex was (− 46.2 ± 0.2 kcal/mol) compared to (− 44.1 ± 0.2 kcal/mol) for the reference complex). The most favourable interaction was Van der Waals interaction with an average around − 63.1 ± 0.22 kcal/mol for compound 5—Eg5 and an average around − 57.8 ± 0.12 kcal/mol for the reference complex. The electrostatic interaction for compound 5—complex was a favourable interaction with a value of − 21.0 ± 0.12 kcal/mol, which was positive value for the reference compound 12.4 ± 0.1 kcal/mol. Both systems showed the same non-polar interactions of − 6.8 ± 0.1 kcal/mol (Table 6).

**Table 6:** Binding free energies and its components, compound 5, reference compound complexes

Energy term	Reference compound—complex Kcal/mol	Compound 5-Eg5 complex Kcal/mol
Evdw	− 57.8 ± 0.1	− 63.1 ± 0.2
Eele	12.4 ± 0.1	− 21.0 ± 0.1
Egb	8.1 ± 0.1	44.8 ± 0.2
ΔEsurf	− 6.8 ± 0.1	− 6.8 ± 0.1
Δ G	− 44.1 ± 0.2	− 46.2 ± 0.2

Energies are calculated in kcal/mol with corresponding standard errors of the mean

**Fig. 12:**
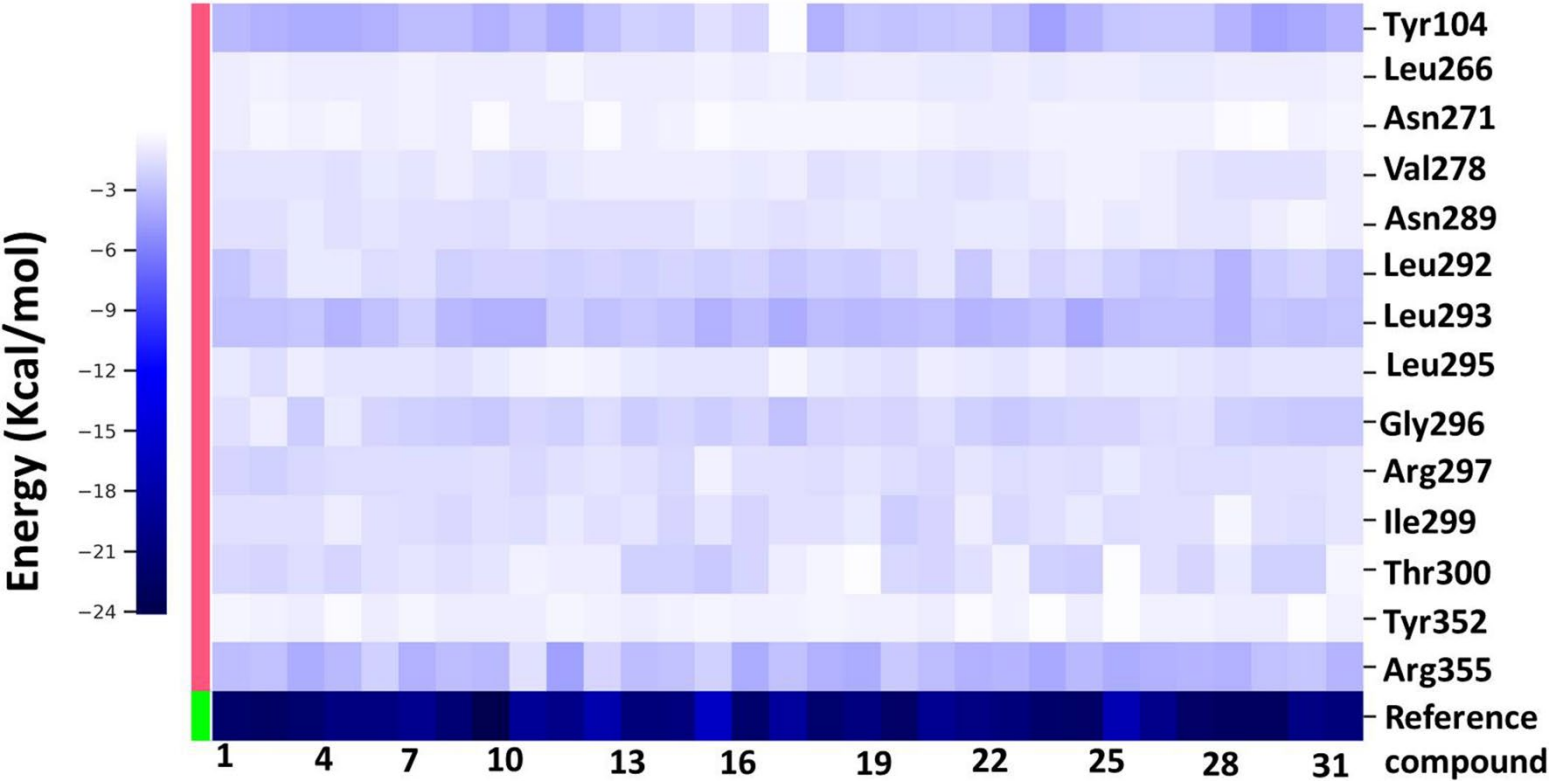
MM-GBSA free energy decomposition of EG5 residues

Further, per-residue decomposition analysis of compound-5 complex was computed to determine the contributions of residues to the binding free energy (Fig. 12). It was found that Tyr104 and Tyr352 contributed with an average energy of around − 5 kcal/mol and − 3 kcal /mol and this can be explained by the face-face aromatic interactions with compound 5, and H-bond with Tyr352. Furthermore, Asn289, Gly296 and Thr300 contributed well to the binding affinity; the three residues formed H-bonds in high incidences, more than 50%. The hydrophobic residues Leu292, Leu293 and Leu295 formed strong hydrophobic interactions with compound 5 with proportion more than 90%. In addition, the two residues Asn271 and Arg277 formed polar interactions with compound 5, around 60% occurrence and contributed significantly to the binding free energy. It could be concluded that the key residues Tyr104, Asn289, Leu292, Leu293, Gly296, Arg297, Thr300 and Arg355 which formed the allosteric site (α4/α6/L11) contributed to the binding free energy of compound 5 with an of average − 5, − 3, − 4, − 5, − 4, − 3, − 4 and − 6 kcal/mol. Interestingly compound 5 showed a slightly more favourable binding energy than that for the reference compound, which may be attributed to the continuous H-bonds with the key residues Asn289, Gly296, Tyr352 and Arg355 and the pi-pi interactions with Tyr104, which resulted in significant contribution ≥ − 3 kcal/mol to the binding free energy of compound 5.

## Conclusion

Eg5 is an attractive anti-cancer target, the inhibitors of Eg5 proteins are divided into two main groups. Group 1 (α2/loop L5/helix α3 pocket) inhibitors and group 2 (α4 / α6/ L11 pocket) inhibitors. Many of Eg5 protein inhibitors have been studied, however only few have been investigated for cancer treatment in clinical trials. Ispinesib is one of the most promising Eg5 inhibitors, it belongs to group 1 and binds to the allosteric binding site (α2/loop L5/helix α3 pocket) but it suffers from cellular resistance. As a result, novel compounds that target (α4 /α6 / L11 pocket) were developed such as GSK-1 and GSK-2, and unlike Ispinesib they do not face cellular resistance. However, GSK-1 and GSK-2 were not clinically successful and therefore there is an urgent need for novel scaffolding, which acts as anti-cancer agents. In this study we used computational techniques to identify novel Eg5 inhibitors that target the α4/α6/l11 pocket. Firstly, the ligand-based virtual screening similarity search was applied to filter out the Zinc drug library of 200 000 compounds then physicochemical investigations were applied, which resulted in 400 compounds that are applicable. Consensus docking using MOE and AutoDock of the 400 compounds was performed then followed by consensus scoring and 80 compounds passed. The virtual inspections resulted in 6 hits, namely ZINC43068613, ZINC1300905, ZINC587060476, ZINC2639178, ZINC06444857 and ZINC43011180. The anti-Eg5 ATPase activities of the 6 compounds were evaluated at 10 µM, compound 5 (ZINC06444857) showed the best anti-Eg5 ATPase activity and compound 5 disrupted spindles in the mitotic cells giving a phenotype similar to Monastrol. As a result, compound 5 was selected for further investigations, and its IC_50_ against Eg5 ATPase enzyme was 2.37 ± 0.15 µM. The molecular dynamics were performed to predict the mode of action of compound 5 and confirm its stability within the allosteric pocket. The results indicated the importance of residues Tyr104 and Tyr352 for the activity. The novel scaffold of compound 5 (8-(3-(1H-imidazol-1-yl) propylamino)-3-methyl-7-((naphthalen-3-yl) methyl)-1H-purine-2, 6 (3H, 7H)-dione) and the potential activity of compound 5 provides a good starting point for further pharmaceutical chemistry development and hit optimization. More biological and computational studies are still required to optimize the activity and safety profile of compound 5.

## Supplementary Information

Below is the link to the electronic supplementary material.

Supplementary file1 (DOCX 208 KB)
