# Supplementary material for: Identifying and characterising promising small molecule inhibitors of kinesin spindle protein using ligand-based virtual screening, molecular docking, molecular dynamics and MM‑GBSA calculations
Source: J Comput Aided Mol Des. 2024 Apr 1;38(1):16. doi: 10.1007/s10822-024-00553-5 (PMC10982093; doi:10.1007/s10822-024-00553-5)
Supplement: Supplementary file 1 — Supplementary file1 (DOCX 208 KB) [file 10822_2024_553_MOESM1_ESM.docx]

| **A)**  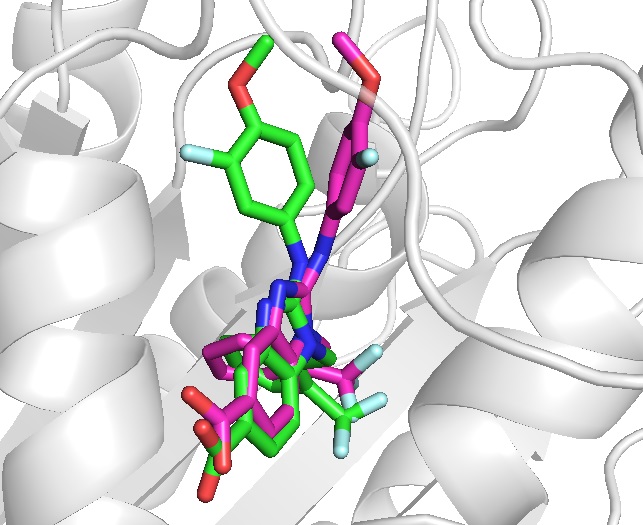 | **B)**  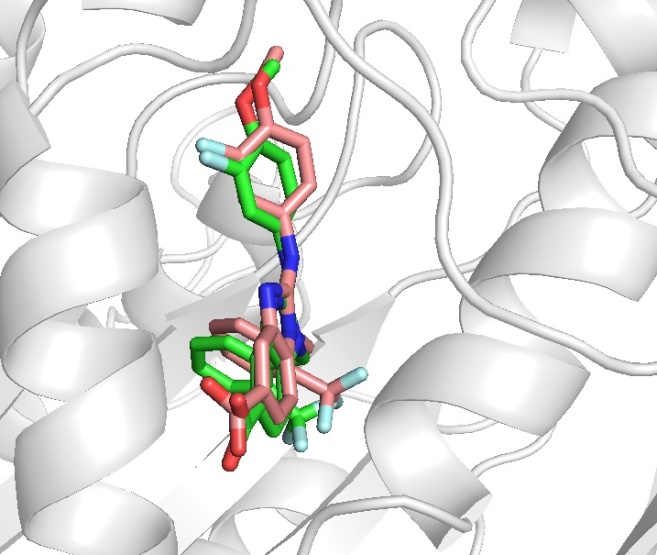 |
| --- | --- |
|  | |

S1) A) Superposition of the initial docked pose of reference compound (green, stick), and the pose (pink, stick) at 1.5Å RMSD. B) Superposition of initial docked pose (green, stick) and the pose (salmon, stick) at 0.5Å.

Table S1 Average RMSD values (Å) of Apo-protein, compound 5 and reference compound complexes

with Eg5 over 200ns of the simulation.

|  | Apo-protein | Compound 5-complex | Reference compound - complex |
| --- | --- | --- | --- |
| Average RMSD (Å) | 3.5±0.2 | 3.0±0.3 | 3.1±0.3 |
| Average RMSF (Å) | 0.33±0.4 | 0.28±0.3 | 0.30±0.3 |
